# Monolithic temperature-insensitive high-*Q* Ta_2_O_5_ microdisk resonator

**DOI:** 10.1515/nanoph-2025-0485

**Published:** 2025-12-05

**Authors:** Zhen Yang, Zheng Zhang, Peng Cheng, Zhe Long, Qi Cheng, Jiaqi Yang, Yu Lin, Bin Fang, Zhongming Zeng, Zhiping Zhou, Ganapathy Senthil Murugan, Rongping Wang

**Affiliations:** Laboratory of Infrared Materia and Devices, Advanced Technology Research Institute, Ningbo University, Ningbo, Zhejiang, 315211, China; Nanofabrication Facility, Suzhou Institute of Nano-Tech and Nano-Bionics, Chinese Academy of Sciences, Suzhou, 215123, China; State Key Laboratory of Advanced Optical Communications Systems and Networks, School of Electrics, Peking University, Beijing, 100871, China; Optoelectronics Research Centre, University of Southampton, Southampton, SO17 1BJ, UK

**Keywords:** tantala waveguide, microdisk resonator, optical cavity

## Abstract

We demonstrate a temperature-insensitive high-*Q* tantalum oxide (Ta_2_O_5_) microdisk resonator fabricated using electron-beam lithography and inductively coupled plasma reactive-ion etching. The microdisks exhibit a loaded *Q*-factor of 4.25 × 10^5^ at 1,550 nm, which more than doubles (∼9.3 × 10^5^) following thermal annealing at 600 °C. Remarkably, the temperature-dependent resonant wavelength shift is suppressed to less than 10 pm/°C across a broad 100 nm bandwidth. Furthermore, the resonators maintain high optical stability under elevated input powers, with no observed degradation in optical properties such as extinction ratio or *Q*-factor. The combination of high *Q*-factors and exceptional thermal stability positions the Ta_2_O_5_ microdisk resonators as a promising platform for integrated photonic device applications, including on-chip narrow-linewidth lasers and precision sensing.

## 1 Introduction

Optical microresonators with high-quality factors (high-*Q*) and low optical losses are fundamental building blocks for diverse integrated photonic applications [1], [2], including nonlinear optics [3], precision biochemical sensing [4], and on-chip lasers [5]. These microcavities extend the photon lifetimes and tightly confine lightwaves within small volumes, significantly enhancing the intensity of the optical resonance fields. This enhancement improves device performance and enables novel photonic functionalities [6]. Recent advances have demonstrated high-*Q* microcavities on numerous material platforms, such as silicon (Si) [7], silicon nitride (Si_3_N_4_) [8], tantalum oxide (Ta_2_O_5_) [9], lithium niobate/tantalate (LiNbO_3_/LiTaO_3_) [3], [10], [11], [12], silicon carbide (SiC) [13], and III-V semiconductors [14]. However, the resonant wavelengths of high-*Q* microresonators are highly sensitive to environmental temperature fluctuations. This instability poses a critical challenge for applications requiring temperature-independent operation, such as dense wavelength division multiplexing (DWDM) systems and frequency-stabilized microlasers. A key limitation lies in the difficulty of simultaneously achieving both high-*Q* factors and thermal stability in existing microresonators. In previously reported adiabatic microcavity devices, materials with negative thermo-optic coefficients are used as cladding, and the waveguide size is reduced to expand the effective interaction area between the light field in the waveguide core and the negative thermo-optic coefficient material, enabling adiabatic operation across the entire waveguide. However, these approaches typically yield large mode volumes and *Q* factors on the order of 10^4^ [15], [16], [17], which significantly degrade performance in practical photonics devices. Especially for on-chip light source applications, achieving thermally stable operation on a single material platform with small mode volume (leading to a stronger interaction between the optical field and the material) without sacrificing the optical performance of the microcavity device is crucial, and further extensive research is still needed.

Among the numerous silicon-compatible materials in photonic research, Ta_2_O_5_ stands out as a promising material for integrated photonic devices due to its attractive features, including a wide transparency window spanning the ultraviolet to infrared (0.28–8 µm) [18], a high refractive index (*n*_0_ = 2.05 at 1,550 nm) relative to SiO_2_ [19], a relatively high nonlinear refractive index (7.2 × 10^−19^ m^2^/W) [20] – approximately three times larger than that of silicon nitride – and a low thermo-optic coefficient (5.75 × 10^−6^/K), which is two orders of magnitude smaller than that of silicon (1.34 × 10^−4^/K) [21]. In addition, Ta_2_O_5_ allows straightforward and high-throughput deposition of high optical quality thin films onto diverse substrates at room temperature. To date, Ta_2_O_5_-based photonic devices have been applied in various fields, such as dielectric metasurface optics [22], nonlinear optics [9], and rare-earth ion doped waveguide amplifiers [23]. Although the thermal stability of Ta_2_O_5_ microring resonators has been characterized [21], [24], [25], the thermal stability performance of Ta_2_O_5_ microdisk cavities has not been reported.

In this paper, we report an athermal, high-*Q* Ta_2_O_5_ microdisk resonator fabricated using electron-beam lithography and inductively coupled plasma reactive ion etching. After annealing, the *Q*-factor of the Ta_2_O_5_ microdisk resonator reaches approximately 9.3 × 10^5^. At the same time, thermal stability across a bandwidth of approximately 100 nm, along with high power-handling stability without degradation in optical performance, is achieved. Synchronized implementation of ultrahigh *Q*-factor and exceptional thermal stability in Ta_2_O_5_ microdisk is key novelty of this work, demonstrating the significant potential of Ta_2_O_5_ microdisk resonators for applications in telecommunications, quantum optics, and precision measurement, where the demand for stable, high-*Q* resonators continues to rise.

## 2 Device fabrication and measurement

**Figure 1:**
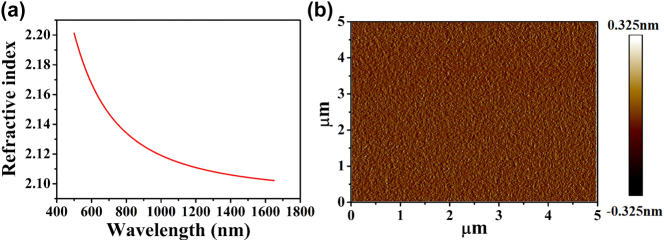
(a) The measured refractive index, and (b) an AFM image of the deposited Ta_2_O_5_ film.

The Ta_2_O_5_ microdisk resonator was fabricated as follows. A 400 nm-thick Ta_2_O_5_ film was deposited via ion beam sputtering (IBS) onto a 4-inch silicon wafer with a 2 µm-thick thermal oxide layer. The metallic Ta target was sputtered with an oxygen flow of 48 sccm and a sputtering power of 400 W in a home-made IBS system. The refractive index dispersion of the deposited film was characterized using a Woollam V-VASE32 ellipsometer and is plotted in Figure 1(a). The surface roughness of the Ta_2_O_5_ film, measured using a Bruker Dimension ICON atomic force microscope (AFM), was determined to be R_q_ (RMS) = 0.08 nm, as shown in Figure 1(b).

**Figure 2:**
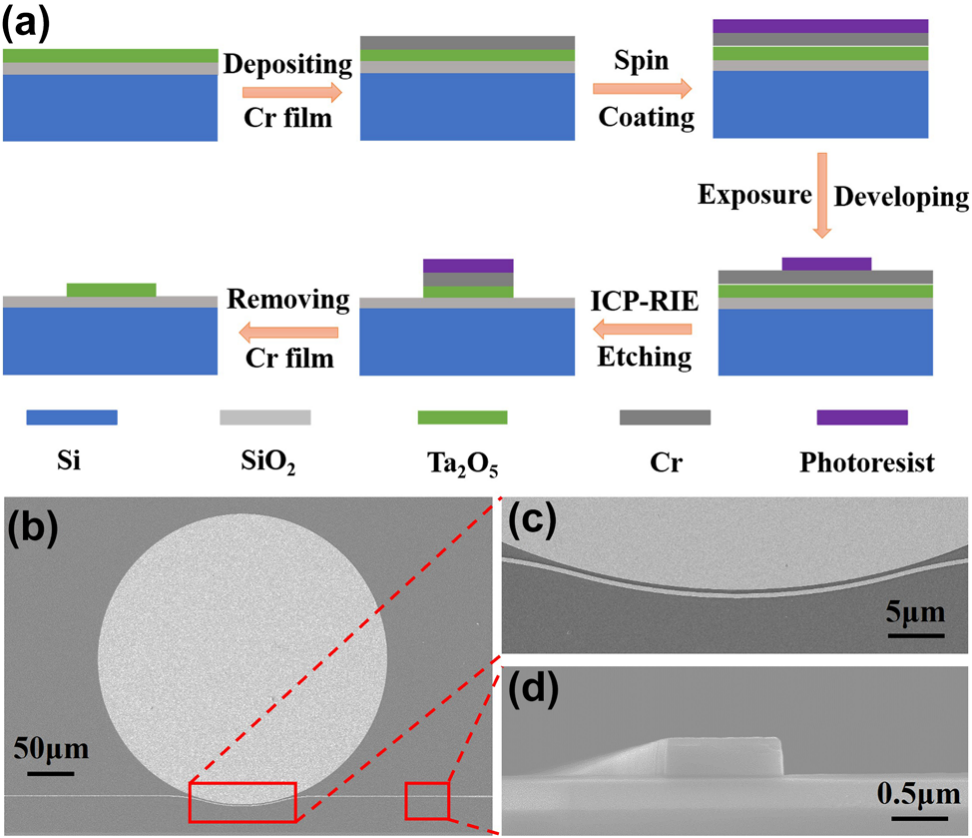
(a) Schematic illustration of the fabrication process flow. (b) SEM image of a Ta_2_O_5_ microdisk resonator with a radius of 150 μm and pulley-type coupler. (c) Magnified SEM image of the coupling region highlighted in (b), showing the evanescent coupling gap between the waveguide and microdisk. (d) Cross-sectional SEM image of a fabricated Ta_2_O_5_ waveguide, with a width of 1,000 nm and a height of 400 nm.

Figure 2(a) illustrates the fabrication process of the Ta_2_O_5_ microdisk resonator. Initially, an 80 nm-thick chromium (Cr) was deposited on the surface of the Ta_2_O_5_ film, serving as a hard mask. Subsequently, the devices were patterned with a 150 nm-thick hydrogen silsesquioxane (HSQ) photoresist using electron beam lithography (RAITH e-LINE Plus). The device structure was transferred to the Cr film through etching with a mixture of Cl_2_ and O_2_ gases. The underlying Ta_2_O_5_ layer was then fully etched using a low-pressure mixture of O_2_, Ar and CF_4_ gases in an inductively coupled plasma reactive ion etching (ICP-RIE) system (ULVAC NE-550). After etching, the device surface was treated with 400 W oxygen plasma for 10 min to remove the etching byproducts, and the Cr hard mask was subsequently removed using a chromium etchant (CR7T).

Figure 2(b) shows a scanning electron microscope (SEM) image of the fabricated microdisk resonator with a radius of 150 μm. The resonators are coupled to an access waveguide to enable optical characterization through transmission measurements. The magnified view of the coupling region, highlighted by the red frame in Figure 2(b) is shown in Figure 2(c). Figure 2(d) presents the cross-sectional SEM of a fabricated Ta_2_O_5_ waveguide. The waveguide has a width (*w*) = 1,000 nm, a height (*h*) = 400 nm, and sidewall angles (*θ*) = 89°. Evidently, the sidewalls are vertical and smooth, demonstrating the high quality of the fabrication process.

In the experiments, light with a sub-MHz linewidth from a tunable laser (Santac TSL-550) was coupled into the bus waveguide end facet via a lensed fiber (SMF28e). On the output side of the waveguide, the transmitted light was collected and directed into a low-noise power meter (Santec MPM210) using a single-mode fiber (SMF). The coupled optical power was maintained at a low level (∼100 µW) to minimize both nonlinear and thermo-optic effects within the Ta_2_O_5_ waveguide. The polarization of the input light was adjusted using a manual fiber polarization controller (Thorlabs FPC526) to excite the fundamental quasi-TE mode of the waveguide. To prevent any thermal drift of the resonator modes, the experimental temperature was fixed at 25 °C using a thermoelectric controller.

## 3 Results and discussion

**Figure 3:**
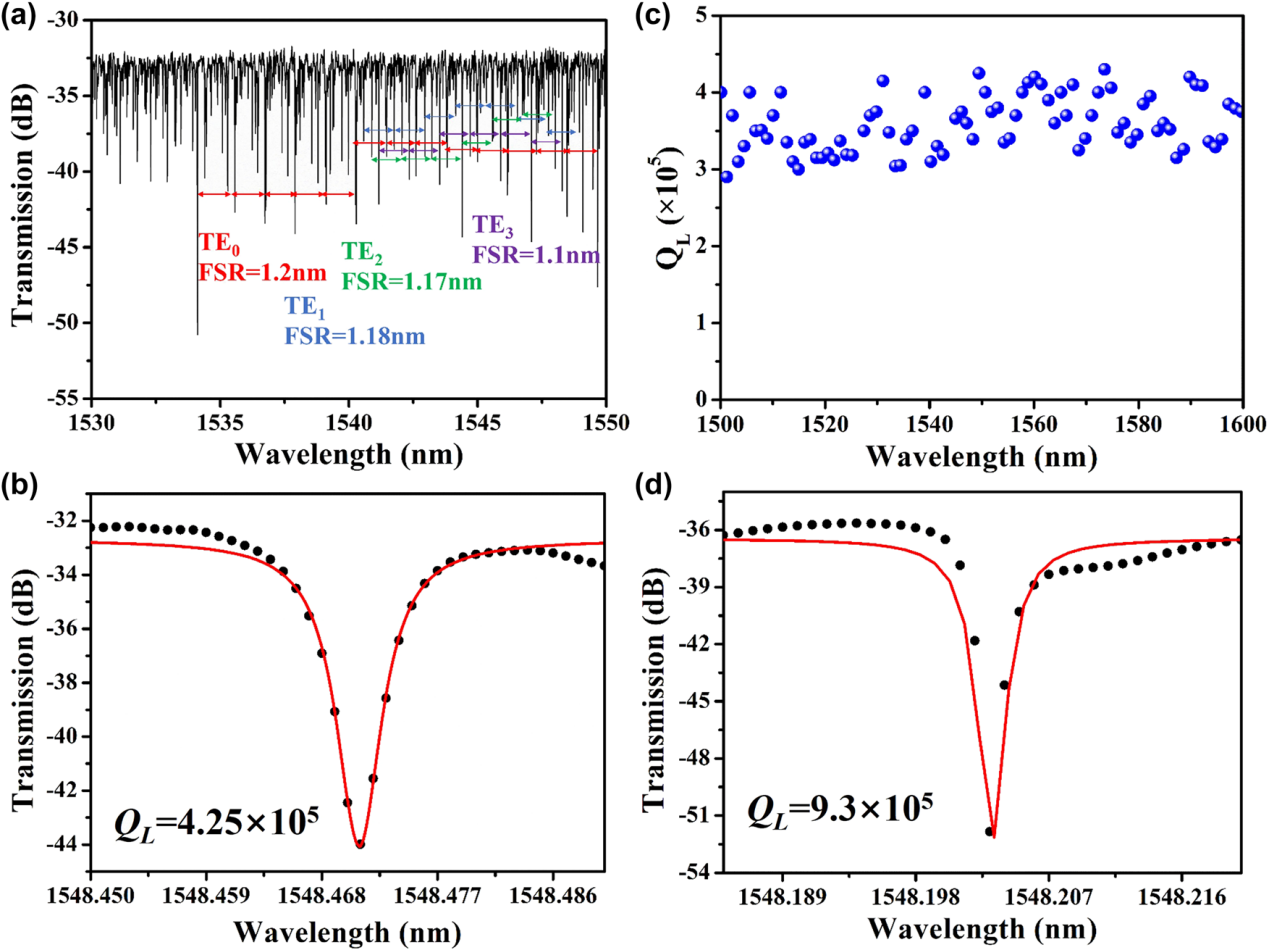
(a) Measured transmission spectrum for the Ta_2_O_5_ microdisk resonator with radius = 150 μm and gap = 490 nm. (b) Measured spectrum (black dots) and Lorentzian fitting (red line) around the resonant wavelength at 1,548.471 nm. (c) Extracted *Q*_*L*_ (blue dot) at each resonant wavelength. (d) Measured *Q* factor of microdisk resonance wavelength at 1,548.2 nm after 600 °C annealing.

Figure 3(a) shows the representative transmission spectrum of the fabricated Ta_2_O_5_ microdisk resonator (radius: 150 µm) measured at room temperature (25 °C). The power of the coupled light was maintained at a low level (∼100 µW) to minimize any nonlinear effects. The microdisk resonator operated in an under-coupled region with a 490 nm coupling gap. Since the input laser coupled to microdisk resonator was adjusted to TE polarization, the resonant mode in the transmission spectrum of the microdisk is primarily dominated by TE modes, with no significant TM modes observed. The Ta_2_O_5_ microdisk in this study has a radius of 150 μm and led to the excitation of high-order TE modes. By analyzing the transmission spectrum, we identify the most prominent resonant peaks as TE_0_, TE_1_, TE_2_, and TE_3_ modes, with corresponding free spectral ranges (FSRs) of 1.2 nm, 1.18 nm, 1.17 nm, and 1.1 nm, respectively. These results are shown in Figure 3(a). The representative resonant peak at 1,548.471 nm for the TE_0_ mode was fitted with a Lorentzian model as shown in Figure 3(b), yielding a full width at half maximum (FWHM, denoted as Δλ) of 3.6 pm. Using the relation *Q* = λ/Δλ, the loaded *Q* factor (*Q*_*L*_) was calculated as ∼ 4.25 × 10^5^ at this wavelength. The intrinsic *Q*-factor (*Q*_*i*_) was determined to be 6.78 × 10^5^ by using the equation $Q_{i}=2Q_{L}/\left(1+\sqrt{T_{0}}\right)$ [19]for under-coupled devices, where *T*_*0*_ (=0.065) is the transmission normalized to the minimum value of the fitting curves at the resonant wavelength.

Figure 3(c) shows the *Q*_*L*_ extracted from all resonances of the TE fundamental mode between 1,500 nm and 1,600 nm. It was found that *Q*_*L*_ -values distribute at around ∼3.5 × 10^5^ in the investigated wavelength range. To the best of our knowledge, this is the first demonstration of high-*Q* Ta_2_O_5_ microdisks with bus waveguide coupling fabricated via electron beam lithography (EBL) and inductively coupled plasma reactive ion etching (ICP-RIE) – in contrast to previously reported high-*Q* Ta_2_O_5_ microdisks fabricated using photolithography-assisted chemical mechanical polishing and coupled via a tapered fibers [19]. To investigate the impact of waveguide sidewall roughness and internal film stress/defects on optical losses, the Ta_2_O_5_ microcavities were annealed at 600 °C for 10 h. As shown in Figure 3(d), the *Q*_*L*_ increased significantly to ∼ 9.3 × 10^5^ in the annealed devices, and the intrinsic *Q* was calculated to be 1.8 × 10^6^. With annealing, the microdisk coupling state gradually shifted towards a critical coupling state. Consequently, the extinction ratio (ER) increased after annealing. Further reduction in optical losses may be achieved by optimizing the ICP-RIE etching parameters and improving the coupling conditions between microdisk and the bus waveguide. Alternatively, single-mode operation and enhanced *Q*-factors could be realized by employing Euler-bent waveguide designs to suppress higher-order modes and scattering losses.

**Figure 4:**
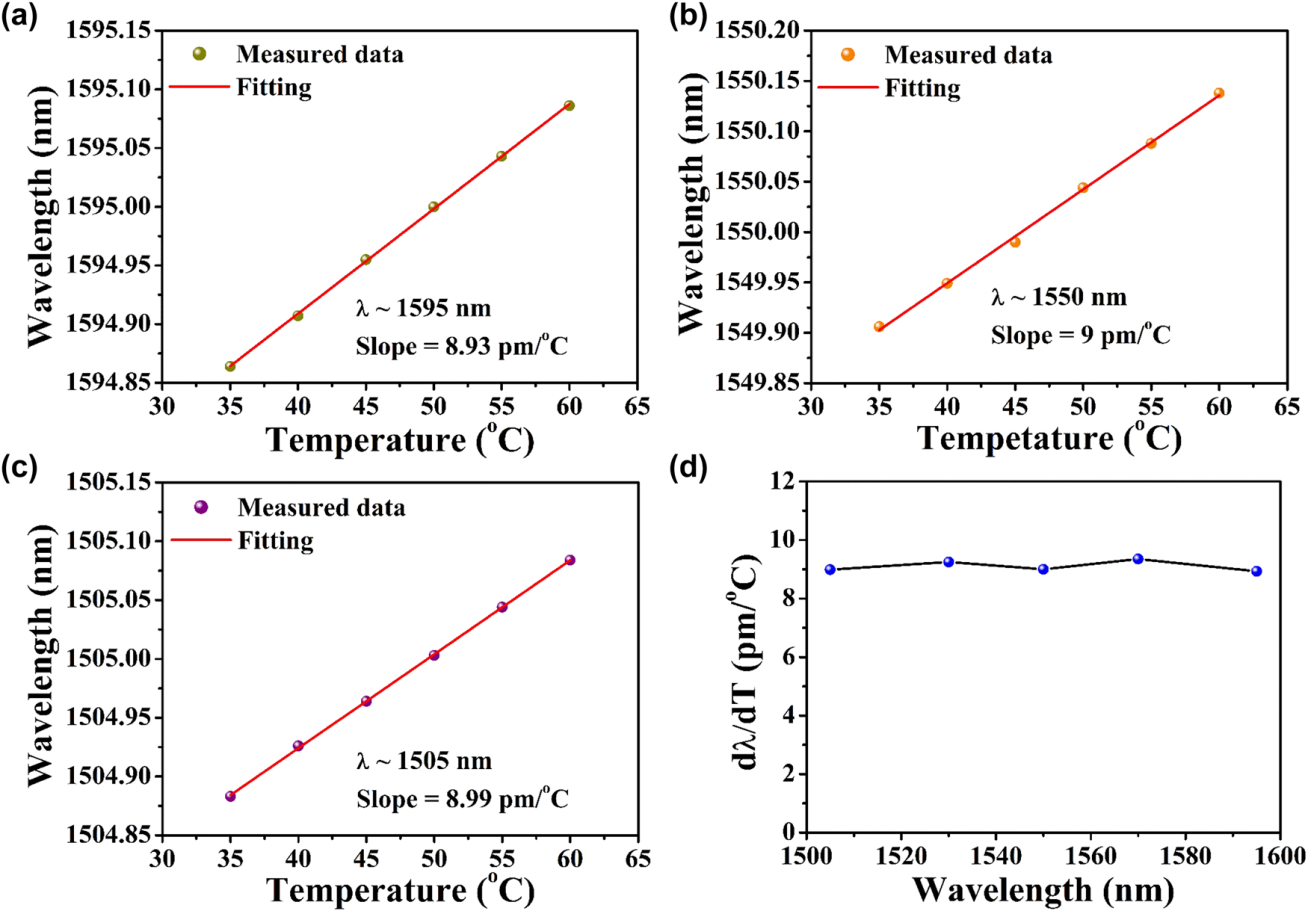
(a)–(c) Fitting of resonance wavelength with increasing temperature at various wavelength ranges around (a) 1,505 nm, (b) 1,550 nm, and (c) 1,595 nm. (d) Resonant wavelength shifts per °C across the entire telecom band.

Thermo-optic properties play a critical role in stabilizing on-chip light sources, particularly in applications such as microcavity-based soliton frequency combs [26] and rare-earth-doped laser systems [27]. To evaluate the thermo-optic response of Ta_2_O_5_ waveguides in the telecommunications band, temperature-dependent TE mode spectral measurements were performed on a Ta_2_O_5_ microdisk resonator. The substrate temperature was varied from 35 °C to 60 °C in 5 °C increments, covering multiple resonant wavelengths. Figure 4(a)-(c) display the linear dependence of the resonant wavelength on temperature at 1,505 nm, 1,550 nm, and 1,595 nm, yielding the resonance wavelength shifts (*dλ*/*dT*) of 8.99 pm/°C, 9 pm/°C, and 8.93 pm/°C, respectively. Figure 4(d) shows the resonant wavelength shift per °C across the entire telecom band, revealing a temperature-dependent shift of less than 10 pm/°C over a ∼100 nm spectral bandwidth.

Table 1 compares the thermal stability of reported athermal microcavities across different material platforms. Microcavities based on materials with high thermo-optic coefficients, such as silicon and lithium niobate, typically require precise geometric engineering and the use of cladding layers with negative thermo-optic coefficients to achieve athermal behaviour. However, these compensatory approaches often compromise optical performance, particularly the achievable *Q*-factors. In contrast, Ta_2_O_5_ offers intrinsic thermal stability due to its naturally low thermo-optic coefficient, enabling broadband athermal behaviour in microdisk resonators while preserving high quality (*Q*) factors (>10^5^). This eliminates the need for complex structural modifications or performance trade-offs.

**Table 1:** Comparison of reported parameters of athermal microcavities based on different material platforms.

Material	Cladding	Structure	d*λ*/d*T* (pm/K)	λ(nm)	*Q*_*L*_	Athermal bandwidth (nm)	FOM^a^ ( $\left|Q_{L}/\Delta \lambda \right|$ )	Ref.
Si	PSQ-LN	Ring	5	1,525	–	–	–	[28]
Si	LFR-372	Ring	0.2	1,550	1.8 × 10^4^	–	9 × 10^4^	[29]
Si	TiO_2_	Ring	6.0	1,310	–	70	–	[30]
Si	TiO_2_	Ring	−1.6	1,548	1.65 × 10^4^	–	1.03 × 10^4^	[15]
Si	EP	Ring	0.5	1,524	–	65	–	[31]
Si	Electro-optic polymer	Ring	2.1	1,550	1.25 × 10^4^	–	0.6 × 10^4^	[32]
Si	As_20_S_80_	Ring	13.95	1,610	2.6 × 10^5^	80	1.86 × 10^4^	[33]
Ta_2_O_5_	Air	Ring	7.8	1,543	5.5 × 10^4^	–	0.71 × 10^4^	[34]
Ta_2_O_5_	SiO_2_	Ring	10.96	1,550	2.29 × 10^5^	–	2.65 × 10^4^	[25]
Ta_2_O_5_	Air	Ring	∼9	1,549	1.84 × 10^6^	–	20.4 × 10^4^	[24]
Ta_2_O_5_	Air	Disk	9	1,550	9.3 × 10^5^	100	10.3 × 10^4^	**This work**
Ta_2_O_5_	SU-8	Disk	3	1,550	3.2 × 10^5^	–	10.7 × 10^4^	**This work**

^a^Higher is better.

The temperature dependent wavelength shift (Δ*λ*) of Ta_2_O_5_ microdisk resonator arises from both the thermal expansion effects and the thermo-optic effects according to Eq. (1) [35]

(1) $$\Delta \lambda =\frac{d\lambda }{dT}=\lambda \left(\frac{1}{n_{\text{eff}}}\frac{dn_{\text{eff}}}{dT}+\frac{1}{R}\frac{dR}{dT}\right)$$

where $\frac{dn_{\text{eff}}}{dT}$ is the effective thermo-optic coefficient, $\frac{1}{R}\frac{dR}{dT}$ is the effective thermal expansion coefficient. However, due to silica’s low thermal expansion coefficient and the thin Ta_2_O_5_ layer in our resonator geometry, material thermal expansion contributions are negligible [17]. From the measured wavelength shift of ∼9 pm/°C at 1,550 nm, we calculated a thermo-optic coefficient (TOC) of ∼ 4.8 × 10^−6^/K for the Ta_2_O_5_ microdisk resonator, which is two orders of magnitude smaller than that of silicon, in excellent agreement with the published values [34], [35].

Since the focus of this study is to simultaneously achieve a high *Q*-factor and low thermal drift, we define a figure of merit (FOM) to compare the performance of our Ta_2_O_5_ resonator with other on-chip cavities. For a fair comparison, we define the FOM given as $FOM=\left|Q_{L}/\Delta \lambda \right|$ , which represents the ratio of the microcavity’s load quality factor to the temperature-dependent wavelength shift. In the equation, *Q*_*L*_ represents the microcavity’s load quality factor, and Δ*λ* represents the temperature-dependent wavelength shift. This FOM does not consider the resonator design and only compares the microcavity’s quality factor and thermo-optical properties across different waveguides. A higher FOM is preferred, implying a higher quality factor and thermal stability of the microcavity. Table 1 summarizes a few representative designs with respect to this FOM.

**Figure 5:**
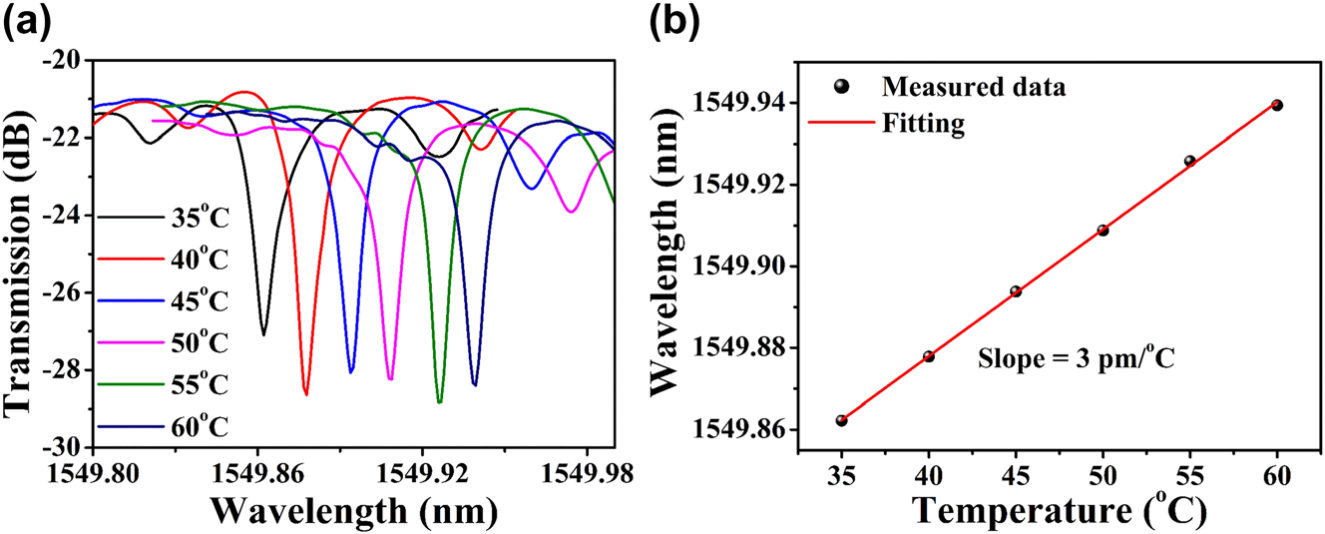
(a) Measured transmission spectra of the Ta_2_O_5_ microdisk resonator at various temperatures with SU-8 cladding, (b) the microdisk resonance wavelength shift with SU-8 cladding as a function of temperature.

To further enhance the thermal stability of the Ta_2_O_5_ microdisk, a 1 μm-thick SU-8 polymer layer was spin-coated onto the device to serve as an upper cladding. The SU-8 polymer, which possesses a negative thermo-optic coefficient (−2.9 × 10^−4^/K) [35], was employed to partially compensate for the positive thermo-optic effect of the Ta_2_O_5_ microdisk. The resonance shift of the microdisk was characterized over a temperature range from 35 °C to 60 °C. As shown in Figure 5(a), the resonance peak at 1,549.8622 nm wavelength was red-shifted to 1,549.9394 nm. Figure 5(b) shows the linear relationship between resonant wavelength and temperature, with a fitted temperature sensitivity of approximately 3 pm/ ^o^C, in contrast to the previous 9 pm/ ^o^C, confirming that SU-8 upper cladding layer significantly improves thermal stability of the device. However, the improvement is limited. Due to the fixed geometry of the microdisk, the majority of the optical mode remains confined within the Ta_2_O_5_ core, and only a small portion interacts with the SU-8 cladding. As a result, the influence of SU-8’s negative thermo-optic coefficient on the overall device stability is modest. The measured *Q*-factor of the SU-8–coated microdisk is approximately 3.2 × 10^5^, lower than that of the air-clad counterpart. This reduction is likely due to unoptimized structural parameters, including cladding thickness, coupling coefficient, and microdisk radius. Here, we mainly characterize the thermal stability of the microdisk working in TE mode, and the thermal stability performance of the microdisk working in TM mode can be further tested later. The TE mode has a higher light field confinement capability than the TM mode, resulting in more light being confined to the Ta_2_O_5_ waveguide core and less light leaking into the SU-8 cladding. Conversely, for the TM mode, more light leaks into the SU-8 cladding. Therefore, under the same external temperature and input power, Ta_2_O_5_ microdisks operating in the TM mode will exhibit superior thermal stability. Future work should focus on engineering the mode confinement to balance the optical field overlap between materials with positive and negative thermo-optic coefficients through carefully designing the SU-8 cladding thickness and the microdisk’s radius and height. By integrating careful microcavity design with optimized fabrication processes, it will be possible to realize high-*Q* microresonators operating in the athermal regime, thereby enhancing their suitability for applications such as on-chip optical frequency comb generation.

In addition to the previously discussed characteristics, we further investigated the impact of high optical power on the stability of the Ta_2_O_5_ microcavity. Specifically, when the microcavity operates at its resonance wavelength, constructive interference leads to a buildup of intracavity power. In such cases, any linear or nonlinear absorption within the resonator can induce localized heating, which in turn may affect the optical properties of the device. Certain material platforms, such as Si [16] and SiC [36], are known to exhibit significant linear and nonlinear absorption under high-power operation, potentially degrading device performance. To evaluate whether similar effects occur in the Ta_2_O_5_ microdisk resonator, we used a tunable laser source to perform wavelength scans while monitoring the corresponding transmission spectra.

**Figure 6:**
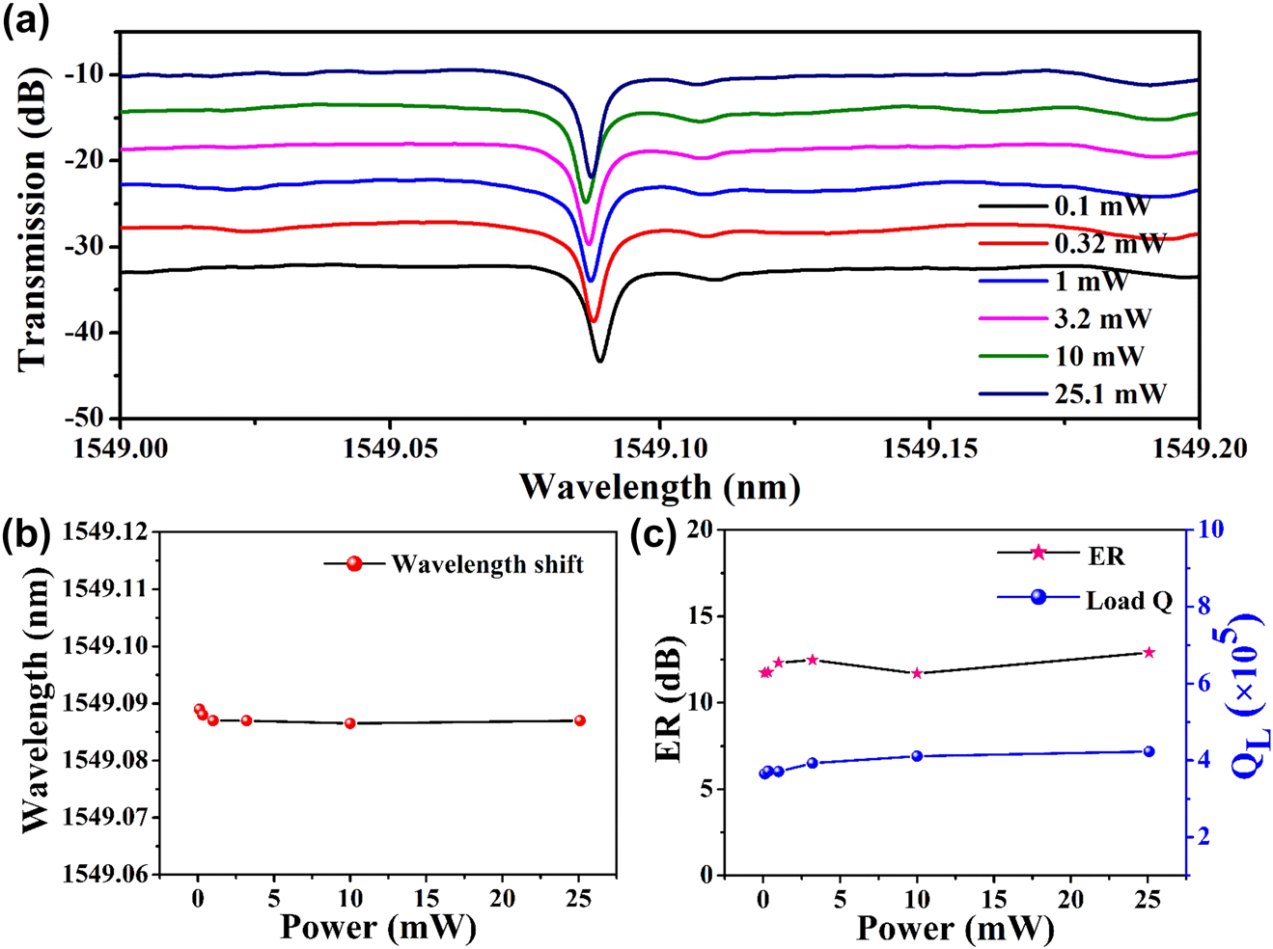
(a) Power-dependent transmission spectra of Ta_2_O_5_ microdisk resonator. (b) Extraction of the resonance peak position at different input power in (a). (c) Changes in the extinction ratio (ER) and loaded *Q* (*Q*_*L*_) of the resonance peak at ∼ 1,549.09 nm in (a) at various input powers.

Figure 6(a) presents the power-dependent transmission spectra of the Ta_2_O_5_ microdisk resonator for coupled input powers ranging from 0.1 mW to 25.1 mW, the latter being limited by the maximum output power of the laser. Notably, the transmission spectra appear nearly identical across all input power levels. The corresponding resonant wavelengths under varying power conditions, extracted from Figure 6(a), are plotted and fitted in Figure 6(b). The results show that the resonance peak position remains virtually unchanged as input power increases. In addition, the *Q* factors and extinction ratios (ER) associated with each resonance peak were extracted and are shown in Figure 6(c). The analysis indicates that neither the *Q*-factor nor the ER is significantly affected by increasing optical power. The *Q*-factors are consistently maintained around ∼4 × 10^5^, and the ER remains approximately 13 dB. The energy buildup in the Ta_2_O_5_ microdisk cavity primarily results from the field enhancement factor (FE). The field enhancement factor describes the ability of the cavity to amplify the local electric field amplitude. The power enhancement factor (*M*) describes the enhancement factor of the optical power stored or circulating within the cavity relative to the incident optical power. Because optical power is proportional to the square of the electric field intensity, the power enhancement factor is numerically equal to the square of the field enhancement factor [37]. At the resonance wavelength, the buildup factor can be estimated by [37], [38]:

(2) $$FE=\left|\frac{\kappa }{1-\tau \exp\left(\alpha L/2+j\kappa L\right)}\right|$$

where a is the round-trip amplitude transmission, *κ* and *τ* are coupling coefficient and transmission coefficient, respectively. They are assumed to satisfy *κ* ^2^ + *τ* ^2^ = 1, and can be calculated using equation $T_{0}={\left(a-\tau \right)}^{2}/{\left(1-a\tau \right)}^{2}$ , here *T*_*0*_ is the fraction of transmitted optical power at the resonance wavelength. *L* is physical length of the microdisk resonator. Based on this, the field enhancement factor in the Ta_2_O_5_ microdisk cavity is estimated to be approximately 22, indicating that the intracavity power at resonance is amplified by a factor of 484 compared to the input power. This observed stability confirms that Ta_2_O_5_ microcavities exhibit exceptional thermal insensitivity and negligible nonlinear absorption, even under conditions of high intracavity energy. These properties make Ta_2_O_5_ a highly promising material platform for developing thermally stable, high-performance on-chip integrated light sources.

## 4 Conclusions

In summary, we have demonstrated a high-*Q*, athermal Ta_2_O_5_ microdisk resonator fabricated using e-beam lithography and inductively coupled plasma reactive ion etching. The fabricated microdisk resonator with a radius of 150 µm exhibits a loaded *Q*-factor of 4.25 × 10^5^ for the TE fundamental mode at 1,550 nm. After thermal annealing, the loaded *Q*-factor more than doubles, reaching approximately 9.3 × 10^5^, with an intrinsic *Q*-factor as high as 1.8 × 10^6^. Notably, the temperature-dependent resonant wavelength shift of the Ta_2_O_5_ microdisk remains below 10 pm/^o^C across the entire telecom band, spanning a ∼100 nm bandwidth. Furthermore, power-dependent transmission measurements reveal no observable shift or distortion in resonant wavelengths, *Q*-factors (4 × 10^5^), or extinction ratios (∼13 dB), even at input powers up to 25.1 mW. These results confirm the exceptional thermal insensitivity and low nonlinear absorption of Ta_2_O_5_ microdisks under high intracavity power conditions. The combination of ultrahigh *Q*-factors and superior thermal stability establishes Ta_2_O_5_ as a highly promising material platform for integrated, narrow-linewidth on-chip lasers [35]. Future work will focus on optimizing thermal compensation techniques, refining fabrication processes, and integrating these microresonators into scalable photonic circuits. This study represents a significant step forward realizing thermally robust, high-*Q* photonic devices, enabling new opportunities in precision metrology, quantum optics and integrated photonic systems.
